# BackMov: Individualized Motion Capture-Based Test to Assess Low Back Pain Mobility Recovery after Treatment

**DOI:** 10.3390/s24030913

**Published:** 2024-01-31

**Authors:** Fernando Villalba-Meneses, Cesar Guevara, Paolo A. Velásquez-López, Isaac Arias-Serrano, Stephanie A. Guerrero-Ligña, Camila M. Valencia-Cevallos, Diego Almeida-Galárraga, Carolina Cadena-Morejón, Javier Marín, José J. Marín

**Affiliations:** 1 IDERGO (Research and Development in Ergonomics), I3A (Instituto de Investigación en Ingeniería de Aragón), University of Zaragoza, C/Mariano Esquillor s/n, 50018 Zaragoza, Spain; jmarinbone@unizar.es (J.M.); jjmarin@unizar.es (J.J.M.); 2 School of Biological Sciences and Engineering, Yachay Tech University, Hacienda San José s/n, San Miguel de Urcuquí 100119, Ecuador; paolo.velasquez@yachaytech.edu.ec (P.A.V.-L.); aarias@yachaytech.edu.ec (I.A.-S.); stephanie.guerrero@yachaytech.edu.ec (S.A.G.-L.); camila.valencia@yachaytech.edu.ec (C.M.V.-C.); dalmeida@yachaytech.edu.ec (D.A.-G.); 3 Department of Design and Manufacturing Engineering, University of Zaragoza, C/Mariano Esquillor s/n, 50018 Zaragoza, Spain; 4 Centro de Investigación en Mecatrónica y Sistemas Interactivos—MIST, Universidad Tecnológica Indoamérica, Quito 170103, Ecuador; cesarguevara@uti.edu.ec; 5 School of Mathematical and Computational Sciences, Yachay Tech University, Hacienda San José s/n, San Miguel de Urcuquí 100119, Ecuador; ccadena@yachaytech.edu.ec

**Keywords:** low back pain, minimal detectable change, deep oscillation therapy, inertial measurement unit, range of motion

## Abstract

Low back pain (LBP) is a common issue that negatively affects a person’s quality of life and imposes substantial healthcare expenses. In this study, we introduce the (Back-pain Movement) BackMov test, using inertial motion capture (MoCap) to assess lumbar movement changes in LBP patients. The test includes flexion–extension, rotation, and lateralization movements focused on the lumbar spine. To validate its reproducibility, we conducted a test-retest involving 37 healthy volunteers, yielding results to build a minimal detectable change (MDC) graph map that would allow us to see if changes in certain variables of LBP patients are significant in relation to their recovery. Subsequently, we evaluated its applicability by having 30 LBP patients perform the movement’s test before and after treatment (15 received deep oscillation therapy; 15 underwent conventional therapy) and compared the outcomes with a specialist’s evaluations. The test-retest results demonstrated high reproducibility, especially in variables such as range of motion, flexion and extension ranges, as well as velocities of lumbar movements, which stand as the more important variables that are correlated with LBP disability, thus changes in them may be important for patient recovery. Among the 30 patients, the specialist’s evaluations were confirmed using a low-back-specific Short Form (SF)-36 Physical Functioning scale, and agreement was observed, in which all patients improved their well-being after both treatments. The results from the specialist analysis coincided with changes exceeding MDC values in the expected variables. In conclusion, the BackMov test offers sensitive variables for tracking mobility recovery from LBP, enabling objective assessments of improvement. This test has the potential to enhance decision-making and personalized patient monitoring in LBP management.

## 1. Introduction

Low back pain (LBP) is the main cause of absenteeism and disability in industrialized societies. Prolonged duration of LBP can significantly affect quality of life due to biomechanical alterations, such as movement alteration, muscular compensation, pustular change, joint degeneration or core dysfunction, reducing the ability to perform daily activities [1]. LBP is an extremely common symptom worldwide and occurs in all age groups, from children to the elderly population [2]. Approximately 10–20% of patients develop chronic LBP, defined as pain and disability persisting for more than 12 weeks [3]. Therefore, there is a clear necessity of reducing the disability caused by this problem; hence, efficient methodologies are needed to improve the function of people suffering these problems.

Traditional physiotherapy treatments have proven to be effective in enhancing function and reducing disability in patients with chronic LBP. Therefore, graded activity or exercise programs that focus on improving function and preventing disability are recommended as primary treatment strategies [4]. Consequently, there is a heightened focus on physical treatments that allow self-management, with less emphasis on pharmacological and surgical interventions [4].

Analyzing how a patient responds to a therapeutic approach by administering pre-treatment and post-treatment tests can help determine whether this should be modified, substituted or discontinued [5,6]. In this regard, some researchers propose the use of tools and indicators to assess the minimal detectable change (MDC) in order to evaluate and ensure actual response to treatments in clinical practice [5,6]. The use of these kinds of tools could reduce or facilitate the monitoring of the effect of treatments without the need to constantly test patients with questionnaire-based measures [7].

The MDC is the minimum quantity of change that can be detected in order to be considered “real” instead of that resulting from potential measurement error [5]. This index represents the variability of the measures of each variable. If a change of one variable is detected and it is lower than its MDC value, it would not be considered statistically significant, since it is lower than the variability of the test [8]. The MDC is important for clinical decision-making because it can provide a threshold value for therapists, clinical therapists, and clinical researchers to determine whether the results represent a real change or reflect intrinsic variability of measurement [9].

The integration of MDC into therapeutic strategies such as DOT or traditional physiotherapy treatment facilitates the evaluation of patient progress. Consequently, physicians are able to monitor patient improvement to ensure that the administered treatments or therapies are yielding satisfactory outcomes [6]. Currently, assessing improvements is normally conducted through qualitative techniques after treatment, either by observation of body movements or through interviews with the patient, as is used for assessing the state of patients before treatment [10]. As exemplified by Marín et al. [10,11], the integration of inertial measurement units (IMUs) for movement analysis via full-body motion capture (MoCap) into rehabilitation assessment based on medical examination is feasible. This is mainly because IMU-based technologies are amongst the most prevalent methodologies employed for MoCap.

MoCap provides information about spatio-temporal and kinematic variables [10,11,12]. These variables are particularly useful for monitoring the progress of patients with musculoskeletal disorders and can offer many opportunities in the field of rehabilitation to aid decision-making using measurements before and after treatment, intervention, or therapy [5,10,11,13,14]. IMUs are electronic devices that capture motion through signal processing of output data from various embedded sensors (accelerometers, gyroscopes, and magnetometers) [15,16,17]. In addition, IMUs have become particularly important because they do not require external cameras and can be embedded in wearable technology [6,18].

Despite the ostensibly broad applicability of clinical movement analysis [19,20], its comprehensive integration into routine clinical practice encounters certain hurdles, most notably the complexity of data analysis stemming from measurement processes. This necessitates the development of strategies for the automatic and consistent processing of the spatiotemporal and kinematic variables information that is generated [10,12]. A particular requirement is the standardization of methods to facilitate a comparative analysis of variables generated from two distinct measurement sessions, for instance, those conducted pre-and post-treatment, or at different junctures during the rehabilitation process [11]. Furthermore, the incorporation of MoCap-based tests needs to address the inherent heterogeneity among patients in routine clinical rehabilitation practice. This entails surmounting the challenge of carrying out individual patient assessments, tracking intra-patient session data over time, individually managing recovery trajectories, and comparing the efficacy of treatments across diverse patient profiles [11].

This study introduces the BackMov test, leveraging inertial MoCap sensor technology for comprehensive lumbar movement analysis. Crafted to objectively track patient progress during rehabilitation, the BackMov test involves two pivotal measurement sessions: pre- and post-treatment. The primary goal is to precisely quantify advancements or setbacks in patients with low back pain (LBP) post-rehabilitation, establishing a correlation between positive changes in kinematic variables of lumbar movements and recovery after therapy. To validate the test’s reliability, a test-retest involving three lumbar movements was conducted on 37 healthy volunteers, utilizing the minimal detectable change (MDC) as a statistical benchmark.

To illustrate its clinical relevance, the BackMov test was administered to 30 LBP-diagnosed patients, capturing the same three-segment movement before and after therapy. The resulting data were then compared to evaluations by a specialist physician. Considered as a novel tool, the BackMov test aims to provide a quantifiable assessment of movement recovery in LBP patients post-therapy. By doing so, it can support specialists in determining the efficacy of treatments in restoring mobility, thereby reducing the disability caused by LBP. The BackMov test enhances decision-making and introduces a systematic approach to the management and treatment of LBP, offering tailored patient monitoring to ensure more effective rehabilitation strategies.

## 2. Materials and Methods

### 2.1. Participants and Protocol

In this study, 37 healthy volunteers, both male and female, were recruited to determine the reproducibility of the test using the minimum detectable change (MDC) criterion. A call for volunteers between 18 and 65 years of age was made through social media. The inclusion criteria for participation in this study included having a diagnosis of low back pain (LBP) made by a doctor or specialist and the experiencing of a reduction in mobility or increased difficulty in movement. The exclusion criteria included individuals presenting any disease or disability that may have hindered their movement, as well as those who were high-level athletes. Additionally, individuals engaging in dangerous activities during the study period were excluded, as well as individuals undergoing any specific drug treatment to alleviate pain. To be eligible for participation, volunteers must not have received physiotherapeutic treatment during the previous 6 months. All participants were required to sign an informed consent form and to attend treatment sessions in person. The final consent was signed on 12 March 2023. Following recruitment, patients underwent the MoCap movement test to evaluate flexion–extension, lateralization, and rotation movements. Among these patients, fifteen (7 men and 8 women) received traditional treatment, including massage, exercise, heat therapy, and cold therapy. The remaining fifteen (7 men and 8 women) were treated with the deep oscillation method to analyze their response to an additional treatment different from the conventional one. Moreover, it was important to know how the clinician evaluated the patient. First, a clinical review was performed, including collecting information about the type of work the patient performed, if the patient practiced any sport, if the patient had experienced any impactful events in the last few weeks or months, and if any type of chronic disease was present. Secondly, the clinician observed the entire posterior trunk, detecting anomalies in the whole spine, accompanied by a palpatory examination of the lumbar spine. Third, the treatments were applied. For the conventional treatment, the clinician applied a combination of different treatments that have yielded positive results. First, a heat treatment was applied for 10 min [21], then a magneto machine was applied for another 20 min [22]. Finally, a massage series was performed [23], which was coupled with three William’s exercises (the first three exercise describe in William’s program) [24] consisting of two series of 10 repetitions of each exercise for five minutes. These treatments were used in order to consider their effects in combination, producing a multi-factorial therapy program that could guarantee yielding results in both the short and long run of the experiment [25].

A deep oscillation treatment was also performed as a novel treatment based on research findings on electrostatic fields used in chronic pain treatment [26]. The clinicians asked the patient to lie down on the table and to remove their T-shirt in order to provide a visible lumbar zone for performance of the treatment. Then, heat was applied to the lumbar region for 10 min. Next, talcum powder was applied and deep oscillation treatment was immediately performed for 15 min. Finally, the patient was massaged on the treated area for 5 min. These massages were also coupled with the William’s exercises as described for the previous treatment. Patients had to perform three sessions per week, i.e., in total, to complete 12 treatment sessions.

To evaluate the patient’s physical improvements, the clinicians used the Short Form-36 Physical Functioning scale specific to low back pain pathologies [27].

**Figure 1:**
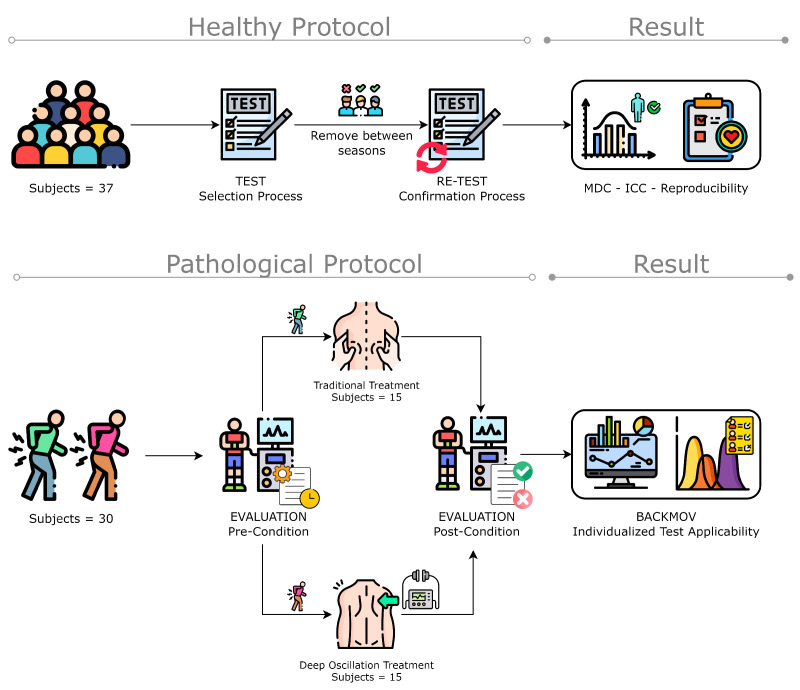
Methodology flowchart of the data acquisition and algorithm implementation for ROM classification (figure designed from Freepik illustrations).

Patient-related information, such as age, BMI, diagnosis, pain level were not included in this document because these variables were not considered when conducting this study. However, information regarding the patients and the procedures performed on each patient is summarized in Table 1. Further details on the status of the patients prior to the treatments can be found in the Supplementary Materials where details of BMI, pain level, age, and a brief description of the situation of the patients is provided. A flowchart of the methodology for ROM classification is presented in Figure 1.

### 2.2. Ethical Statement

The study was conducted in accordance with the Declaration of Helsinki and the protocol was approved by the Ethics Committee for Research on Human Beings of the Pontificia Universidad Católica del Ecuador (PUCE), Ecuador (N° EO-146-2022). Written informed consent was obtained from each participant.

### 2.3. Technology and Instrumentation

**Figure 2:**
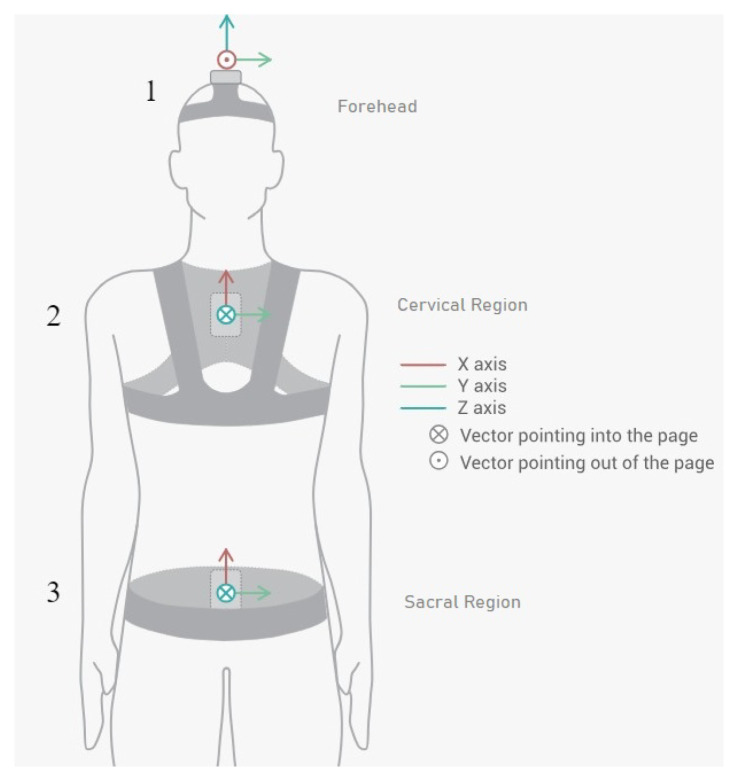
Inertial sensor placements and orientations on patient (sensor 1 in the forehead region, sensor 2 in the cervical region, and sensor 3 in the sacral region).

We employed the Move Human (MH) Sensors MoCap system which was developed by IDERGO (Investigación y Desarrollo en Ergonomía Research Group, V19-07.011, University of Zaragoza, Zaragoza, Spain) using NGIMU (x-io technologies, Bristol, UK). This system relies on inertial measurement units (IMUs) securely positioned on three key areas of the body: sensor 1 is collocated in the superior head region (forehead), sensor 2 is collocated in the cervical region (specifically at C7), and sensor 3 is collocated in the sacral region (at the iliac crest level) (see Figure 2) for comprehensive analysis. This system provides accurate information on the rotations and displacements of each body segment at a frequency of 60 Hz.

The MH-Sensors system enables the visualization and real-time monitoring of movement on a digital representation of a human body, or avatar, which is adjusted to the subject’s body dimensions. In addition, the inertial sensor system houses three types of sensors: accelerometers, gyroscopes, and magnetometers. These signals are combined, enabling the clinician to obtain three rotation angles in the axes of space (as well as the angular velocities and accelerations).

### 2.4. Varirable

**Figure 3:**
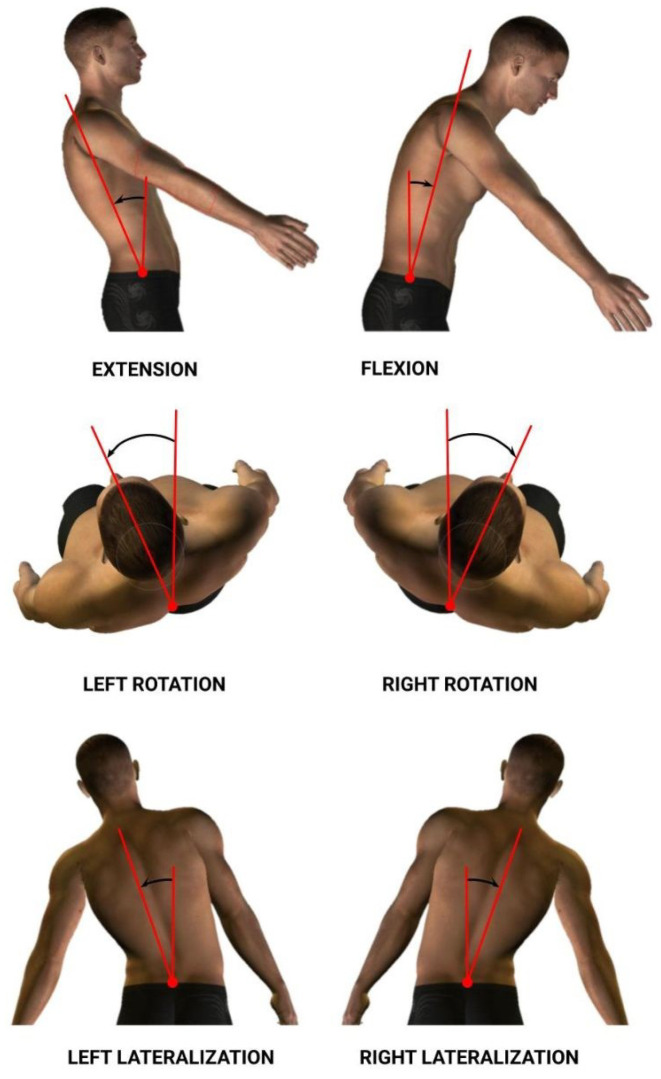
Flexion–extension, right–left rotation, and right–left lateralization lumbar movements.

The dorsal–lumbar movements analyzed to obtain data were flexion–extension (Flex), right–left rotation (Rot), and right–left lateralization (Lat) (See Figure 3). As a result, we acquired information relating to spatiotemporal and kinematic variables. Each variable was calculated for each dorsal–lumbar movement (Flex, Rot, and Lat). Information for the variables considered in the study is summarized in Table 2.

### 2.5. Magnitude-Based Decision (MBD) to Monitor Individuals with LBP

To measure the effects of the treatment on patients, it is necessary to use the statistical approach, magnitude-based decision MBD, in order to compare the effect size with a predetermined threshold. When discussing individual monitoring, it is important to consider that each patient performed six complete cycles during each session (one session prior to the treatments and one session after the treatments; these two sessions were conducted for both types of treatment, traditional treatment and deep oscillation treatment), attempting to reach their maximum range in each exercise in order to obtain the necessary information to evaluate the respective variables needed for assessment. Thus, for individual monitoring of each variable, it was possible to compare two sets of measurements: one from the pre-treatment session (*n*_1_ samples, *X*_1_ mean, and *SD*_1_ standard deviation) and another from the post-treatment session session (*n*_2_, *X*_2_ mean, and *SD*_2_ standard deviation). For this purpose, we used the same discerning method as described in Marin et al. [11], the magnitude-based decision method. This method provides the probability that a change (which is defined by the confidence interval of the difference, CIdiff) exceeds a specific threshold (−δ, +δ) [19], in this case, the MDC [29] (1).

MDC values at 95% confidence were calculated using the following expression:

(1) $$MDC_{95}=1.96\sqrt{2}\mathrm{SEM};\;\mathrm{SEM}={\mathrm{SD}}_{\mathrm{pool}}\sqrt{1-\mathrm{ICC}}$$

In this equation, SD stands for the weighted mean of the standard deviation between test and retest, ICC is the intraclass correlation coefficient, and SEM is the measurement standard error.

In this way, a change is only considered substantial if it overcomes the test’s inherent faults, so that it can be stated that the change observed is real and is not the product of a measurement error. This can be especially helpful to physicians when used in conjunction with clinical data.

**Figure 4:**
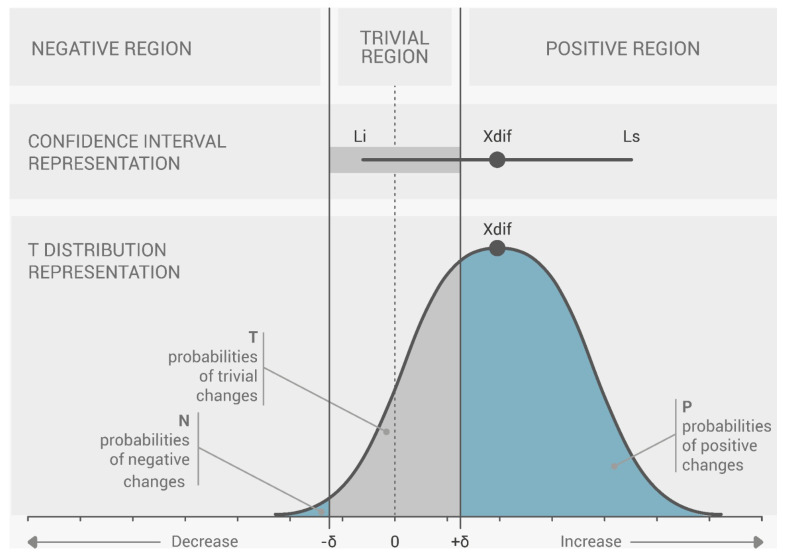
Magnitude-based choices (MBD) threshold: representation of the difference between the pre- and post-series of a single variable. Change within a topic (difference in means between pre- and post-series) is referred to as Xdif. Li: the change’s lower limit; Ls: the change’s upper limit, according to J. Marin, J. J. Marin, T. Blanco, J. de la Torre, I. Salcedo, E. Martitegui [11].

By calculating the CIdiff, we can create a graph for each variable that depicts the threshold (−δ, +δ) and the t-distribution of the change between the pre- and post-series, like the one presented in Figure 4. Analysis of where changes occur with respect to the threshold is made clearer using this depiction. To conduct this analysis statistically, we determined specific domains of probability denoted negative change (N), trivial change (T), and positive change (P). These domains are defined by the proportion of the t area that falls inside; as such, we have a “negative” region (−infty,−delta), a “trivial” region (−delta, +delta), and a “positive” region (+delta,+infty).

In this case, a change is regarded as null or insignificant if it does not surpass the threshold in any direction where the percentages of P and N are both less than 5% (N < 5% and P < 5%). A change is classified as uncertain if both P and N surpass 5% (P > 5% and N > 5%), because it occurs concurrently in both directions. With a predetermined likelihood of change, any further measure of CIdiff can be classified as either positive (increment) or negative (decrement). The probability of change is P when there is an increase in the change, and N when there is a decrease in the change. According to the classification of the likelihood of change, 5 to 25% is considered “unlikely”, 25 to 75% is “possible”, 75 to 95% is “likely”, 95 to 99% is “very likely”, and greater than 99% is considered “extremely likely”.

Apart from the application of the MDC, another important point to observe in the individual analysis is that most changes in the variables are neither necessarily beneficial nor harmful. Increasing or decreasing the magnitude of a particular variable may be beneficial to one patient but harmful to another because LBP affects patients differently. Therefore, the results must be interpreted individually for each patient.

## 3. Results

### 3.1. MDC Index Tables Results from Test-Retest Lumbar Movement Analysis

Table 3, Table 4 and Table 5 are used to show the results of the calculated MDC values from the healthy subjects (absolute value of the MDC at 95% and dimensionless value of the effect size MDC.es at 95%). The tables also summarize the values of the means (μ), standard deviations (SD), and results of the variability through ICC of each of the analyzed variables. Each table summarizes the results for a specific lumbar movement: flexion–extension, rotation, and lateralization, respectively.

### 3.2. Results of the Patient-Level Study

**Figure 5:**
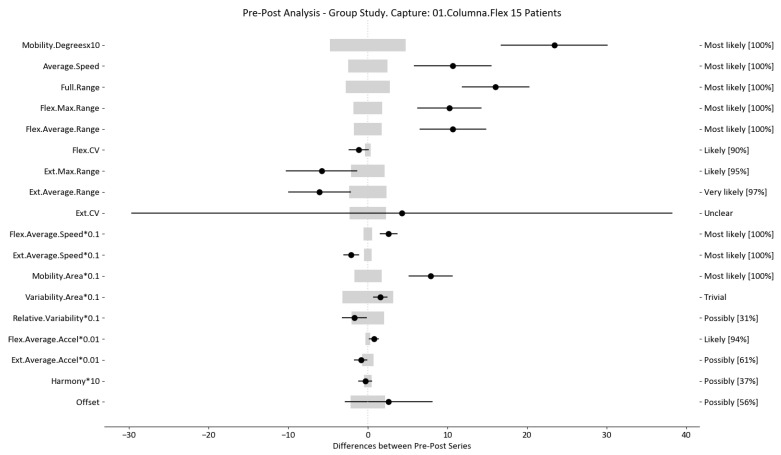
Flexion–extension analysis and confidence interval for DO group. Wide light gray bars: MDC threshold. Thin black bars: confidence intervals of differences.

**Figure 6:**
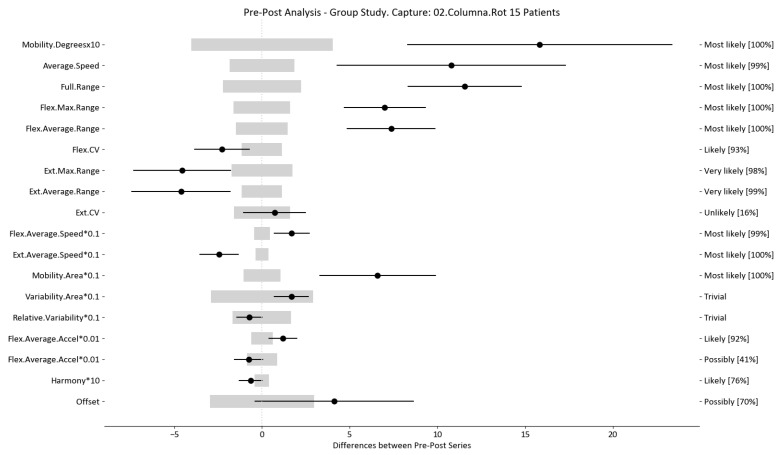
Rotation analysis and confidence interval for DO group. Wide light gray bars: MDC threshold. Thin black bars: confidence intervals of differences.

**Figure 7:**
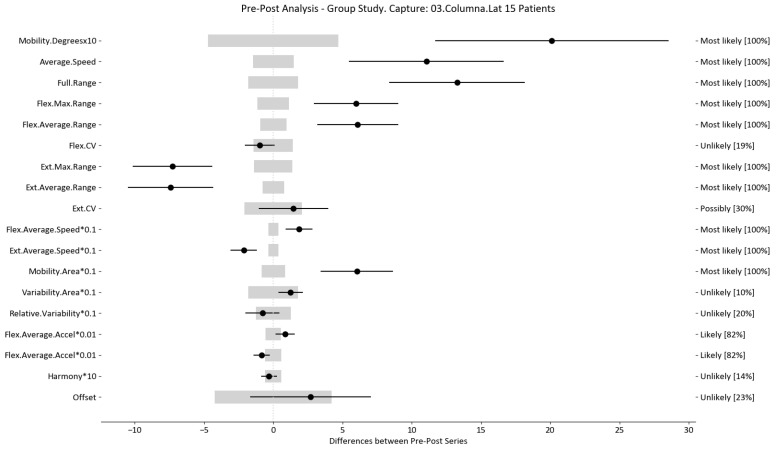
Lateralization analysis and confidence interval for DO group. Wide light gray bars: MDC threshold. Thin black bars: confidence intervals of differences.

**Figure 8:**
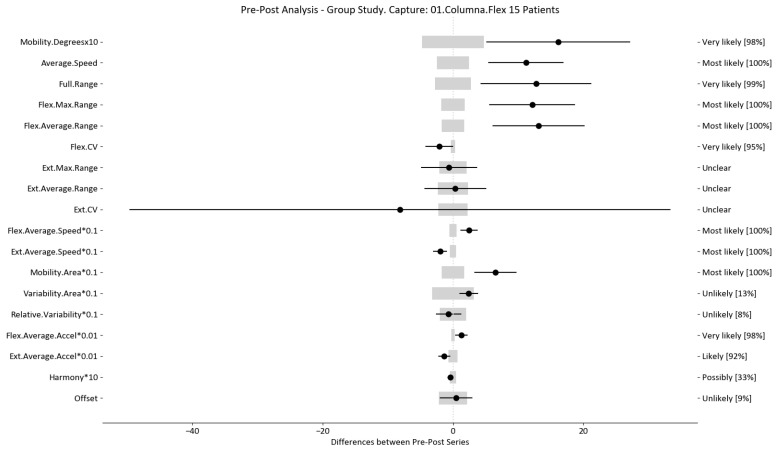
Flexion–extension analysis and confidence interval for TT group. Wide light gray bars: MDC threshold. Thin black bars: confidence intervals of differences.

**Figure 9:**
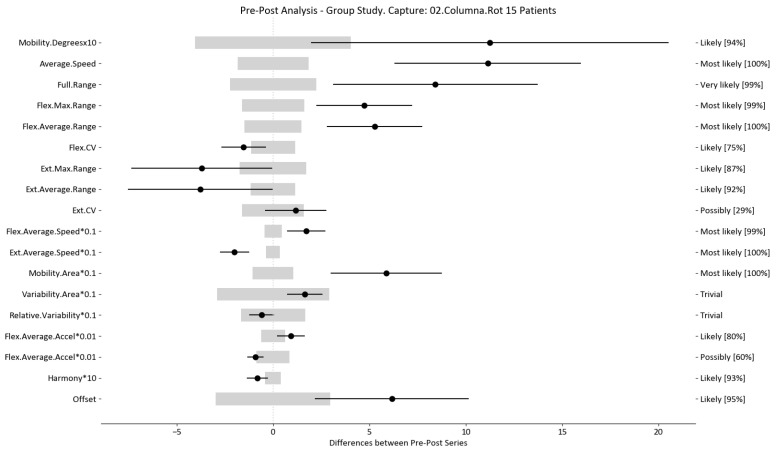
Rotation analysis and confidence interval for TT group. Wide light gray bars: MDC threshold. Thin black bars: confidence intervals of differences.

**Figure 10:**
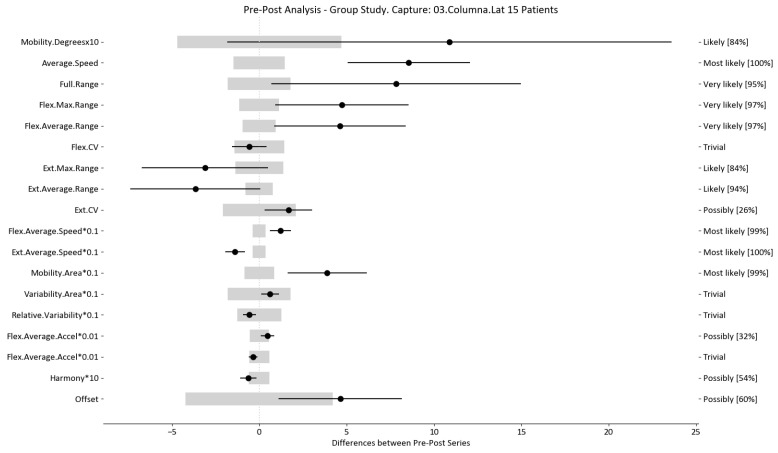
Lateralization analysis and confidence interval for TT group. Wide light gray bars: MDC threshold. Thin black bars: confidence intervals of differences.

The results of this study involved 30 patient-level sets of data summarized into two group analyses corresponding to the two therapy treatments (15 patients each group). In this section, we present the summarized data for the two group analysis. The first group corresponds to the deep oscillation treatment therapy group; its results are summarized in Table 6, Table 7 and Table 8 and Figure 5, Figure 6 and Figure 7; each table and figure is presented for one of the three movements (flexion–extension, rotation, and lateralization, respectively). Table 9, Table 10 and Table 11 and Figure 8, Figure 9 and Figure 10 present data for the second group subject to traditional treatment therapy. These tables include the change between the pre- and post-series of the analyzed variables, the threshold MDC, and the MBD numerical results (i.e., the N, T, and P values; the variables that underwent a significant change are marked with a *). The figures use the confidence interval representation to show the information included in the Tables. The numerical results and the biomechanical interpretation of the single patient analysis are presented as Supplementary Materials in an Excel file (Supplementary Materials S2 and S3).

In relation to the individual data of all patients for both treatments, this information is summarized in the Supplementary Materials. When deciding how to interpret the results, we only considered the changes that exceeded the threshold δ at a level interpreted as ‘very likely’ (above >95% probability); we denominated these as ‘real’ changes.

### 3.3. Results of the Low-Back-Specific Version of the SF-36 Physical Functioning Scale

As established before, this study involved 30 separate patients, each of whom was assessed by a physician specialist using a low-back-specific version of the SF-36 Physical Functioning scale two times; once before the treatment sessions and once after such treatments. In this section, we present the scores determined for the same patients referred to in the previous section (see Table 12). All the scores, as well as the more detailed answers of the patients to the questions from the rest of the patients, are presented in the Supplementary Materials.

## 4. Discussion

To assess the reproducibility in our test-retest results, we evaluated the ICC values, which exceeded 0.7. These results are satisfactory in comparison to those reported in a similar study [30], where use was made of an optical motion capture system to analyze the same movements but in a seated position; in this study only moderate ICC values (0.6–0.7) for most of their variables were obtained. In another study by Megan O’Grady et al. [31], use was similarly made of an IMU system to analyze lumbar movement (flexion–extension, lateral flexion left to right, and rotation). When calculating the reproducibility, these authors obtained values (0.95 < ICC < 1.00) for the kinematics they were evaluating. The values obtained in our study with respect to the same variables were very similar to those reported in Megan O’Grady’s study [31] (ICC > 0.85). Both of these findings indicate the reliability of IMU systems and their potential to assess the kinematics of lumbar movement. Most of the kinetics variables also exhibited high reproducibility (ICC > 0.85), with the exception of the accelerations for the lateralization and rotation movement. When compared to other studies that also dealt with MDC, we note that our results for the ROM were comparable with those obtained in similar studies [32,33]. However, we were unable to find details of previous MDC studies focusing on the velocities of these movements, so we are not able to fully support our findings with respect to these variables.

The utility of the test is determined by the MDC value of the variables, with Table 3, Table 4 and Table 5 (marked with a *) indicating the variables with the lowest MDC ranges and the greatest ICC in our study. Furthermore, based on the statistical application of the MDC technique in a sample of 30 patients, these are the variables most relevant for measuring change at the individual level and, hence, the efficacy of an intervention/treatment. The findings were compared to those of various studies of individuals with LBP, where the most reliable variables are those in which the biggest change was identified in relation to improvement [34,35,36,37,38,39]. As a result, the BackMov test could give clinicians reliable and easy to understand information about the change in a particular characteristic seen in a patient, enabling them to clearly see the improvement or impairment of a patient. The clinician, who can now be aware of these specific characteristics in the patient’s circumstances may find it easier to decide whether or not to continue with a treatment or whether to change it in favor of a treatment to target the specific change that they may have noticed.

For example, we draw attention to the graphs depicted in Figure 5, Figure 6, Figure 7, Figure 8, Figure 9 and Figure 10 with regard to the findings of this study. These graphs were created to graphically and intuitively display the changes observed in the set of variables, making it easier for the physician to recognize the factors that need further consideration and analysis. This information can be useful, as a significant number of guidelines primarily focus on assessing the disability that the LBP may be causing the patient [40]. In relation to this, we saw that the improvement in ROM and velocities could be directly related to the abilities of individuals to perform physical activities. This is supported by the observation that for patients that exhibited a significant change in these variables when responding to the questionnaire conducted by the physicians (see Supplementary Materials S5), their answers showed that most of them were better when performing activities that involved a high degree of movement from their point of view, such as going up the stairs or walking. Furthermore, as the velocities of the patients also seemed to have improved, this could be related to the therapies helping patients to overcome their fear of movement so that they were less hesitant to move, therefore decreasing the overall time it took them to perform an activity [41,42]. Overcoming the fear of movement would correlate with the improvement in these variables with overall improvement in performing daily activities. Furthermore, as movement increases this also helps to reduce muscle tension and stiffness that may have been caused as a result of the individuals trying to move as little as possible to avoid pain [43,44,45]. The results of our MDC comparisons are in line with evaluation of progress in the patients conducted by the specialist physicians using the low-back-specific form of the SF-36 Physical Functioning scale. The results were that both groups showed real changes in the variables that can be associated with disability and thus may influence recovery of LBP patients. In this way, the graphs are intended to make it easier to identify a change in a variable related to disability, thus facilitating monitoring of treatment without the need to keep completing questionnaires to assess progress. Furthermore, if the clinician needs to pursue a more extensive and rigorous analysis, the tables (Table 6, Table 7, Table 8, Table 9, Table 10 and Table 11) contain more detailed information on the variables and the changes in the patients kinematics.

Personalized medicine has become an important goal for health professionals [46]. The response to therapy for conditions like LBP problems can be objectively described through individual patient assessment, so these kinds of tools that reduce the time to assess progression can be good for the future of therapy. Although there seems to be benefit in using the BackMov test for the purposes described, there are certain issues that need to be addressed as they may cause the results to not be reliable if they are not taken into account. A clear issue is the fact that IMUs suffer from integration drift, which involves small errors appearing during the measurement of acceleration and angular velocity that can be progressively combined into larger errors in velocity and angle, which are compounded into still greater errors in position [47]. Another issue that may arise from this test that must be taken into consideration is the effect of noise associated with other variables, including pathology, treatment used between pre- and post-testing, illness progression, and even private personal events [48,49,50,51,52]. Despite the promising results and the potential utility of the BackMov test in assessing lumbar movement recovery in LBP patients post-therapy, it is crucial to acknowledge certain limitations that may impact the interpretation of our findings. One notable limitation is the exclusion criteria employed in participant selection. Excluding individuals with pre-existing diseases or disabilities that might hinder their movement was essential for maintaining homogeneity in the study cohort. However, it is important to recognize that the applicability of the BackMov test to a broader patient population, including those with comorbidities or disabilities, remains to be explored. Another limitation stems from the exclusion of high-level athletes from the study cohort. While this decision aimed to control for the potential influence of exceptional physical fitness on lumbar movement, it has inadvertently limited the generalizability of our findings to this specific subgroup. The BackMov test’s effectiveness in assessing movement recovery in athletes with LBP warrants further investigation to ascertain its relevance and applicability across diverse patient profiles.

Additionally, the exclusion of individuals engaged in dangerous activities during the study period raises questions about the test’s external validity in real-world scenarios where patients may need to resume such activities post-therapy. The BackMov test’s capacity to capture improvements in lumbar movement relevant to occupational or recreational activities involving increased risk remains an avenue for future research. Moreover, the exclusion of individuals undergoing drug-specific treatments to alleviate pain introduces another layer of complexity. Many LBP patients often rely on pharmacological interventions for pain management. The impact of these treatments on lumbar movement and the BackMov test’s sensitivity to changes in patients undergoing drug-specific interventions were not explored in this study. Future investigations should consider incorporating these variables to enhance the test’s applicability in a broader clinical context.

As an important note, while the BackMov test appears to possess great potential to detect when a patient experiences a relevant change in a particular variable (high likelihood of change), the physician should still not only rely on these results for determining whether or not this change is important for treatment. This is significant because, despite the fact that this kind of metric is meant to offer unbiased data in support of a diagnosis, the MDC itself is not a definitive diagnosis and should not be used as the only tool to determine a clinical decision as it is based on merely an association of certain variables with the disability produced by the LBP [53,54,55]. Some of these associations are still in need of further evaluation to be considered directly proportional to the effects of LBP. Furthermore, these values do not account for the psychological aspect that must be addressed when dealing with these kinds of problems [56,57]. Regarding the study’s implications for research and clinical practice, we believe that the use of the BackMov test as a ’logical’ guideline can be the foundation for future patient studies for the evaluation of treatments. However, it is necessary to further develop the rules that allow for more effective qualification of the change detected in the variables. This would further support use of the method in the evaluation of treatments or the monitoring of a patient’s overall recovery based on the premise of disability recovery from improved kinematics of lumbar movements.

## 5. Future Work

To enhance the understanding and potential of this methodology, a more comprehensive and rigorous approach is proposed. In the newly suggested methodology, a control group should be included to observe the progress of LBP patients in the absence of any treatment. Additionally, other therapeutic treatments for the pathology should be incorporated. In future studies, greater attention must be given to the psychological and psychosocial aspects of the disease. Furthermore, the utilization of alternative statistical tools, such as the minimal important difference (MID), should be considered to complement the minimum detectable change (MDC). Determining MID thresholds, alongside MDC, would offer a more valuable means to assess therapies, provide evidence for diagnoses, and facilitate the monitoring of patient recoveries. Lastly, unless more evidence emerges regarding the relationship between disability and kinematic variables or the association of fear of movement with kinematics, a dedicated study focusing on establishing these connections should be planned to support the use of this methodology.

## 6. Conclusions

In conclusion, we can say that the test-retest of the BackMov test that was conducted on a sample of healthy young subjects was able to show excellent reproducibility and validity for variables such as the Max.Range, the Flex.Average.Range, the Ext.Average.Range, the Flex.Average.Speed and the Ext.Average.Speed in all three assessed lumbar movements, thus making the use of IMUs a reliable tool for assessment of range of motion (ROM) evaluation recovery in LBP patients. The assessments made by the expert physician were mainly validated using the objective pre- and post-test outcomes for 30 patients with LBP difficulties. In this approach, the results were demonstrated to be applicable to the evaluated patient, as certain factors were sensitive enough to detect significant changes associated with the progression (improvement, worsening, or no changes) in LBP. From this, we draw the conclusion that the application of clinical lumbar movements analysis based on IMU technology in rehabilitation could be advantageous in this branch of the medical field as an extra tool that may help specialists to assess the extent of recovery of any lost mobility caused by LBP. The results of this study also may contribute to improving medical decision-making and the individualized follow-up of patients; however, more extensive and rigorous studies must be undertaken in order to fully validate the potential for use of these tools.

## Figures and Tables

**Table 1:** Participants.

Group	Male	Female	Total
DOT	7	8	15
TT	7	8	15
Total	14	16	30

Deep oscillation treatment (DOT); Traditional treatment (TT).

**Table 2:** Summary of spatiotemporal and kinematic variables for each dorsal–lumbar movement.

Variable	Description
Max.Range	Overall range of motion in the dorsal–lumbar movement (Maximum extension range plus maximum flexion range).
Max (Min)	Highest value achieved within the range of motion in the dorsal–lumbar movement (Max is for flexion and Min for extension).
Max.Mean (Min.Mean)	Average range of motion in the dorsal–lumbar movement (Max is for flexion and Min for extension).
Coefficient of variation (%) (CV)	Indicator of variability in motion data within the dorsal–lumbar movement, expressed as a percentage.
Average speed (°/s) (Speed)	Average speed of motion in degrees per second in the dorsal–lumbar movement.
Average SpeedUp (°/s^2^) (SpeedUp )	Average acceleration of motion in degrees per second squared in the dorsal–lumbar movement.
Mobility area (AreaMean) *	Displays the total area under the motion curve in the dorsal–lumbar movement, which can provide information about the number of movements performed within a specific timeframe. A larger area indicates greater patient mobility when performing movements.
Variability area (AreaStd) *	Indicates the total area under the variability curve in the dorsal–lumbar region, providing insight into the overall variability of movement within a specific timeframe. It depends on the similarity or resemblance of the different cycles of the resulting graph. A larger area reflects greater variability in the cycles of dorsal–lumbar movement by the patient, indicating less uniformity or regularity in those movements.
Relative variability (coef%) *	Represents a percentage-based measure of relative variability in the dorsal–lumbar region, offering a standardized assessment of variation in motion data.
Harmony *	Evaluates the overall smoothness and coordination of movements in the dorsal–lumbar region. The variable used to calculate the harmony of motion was the Pearson correlation coefficient between the angle and the angular acceleration. A value of “−1” would indicate a maximum correlation, that is, a straight line in the relationship between these two variables.
Offset (°) *	Measures the time delay or phase difference between different movements or segments in the dorsal–lumbar region, expressed in degrees. In this case, these waves are the signals of angle and acceleration of dorsal–lumbar movement.

Variables with * are justified based on the work [28].

**Table 3:** Test-retest results from flexion–extension movement tests. Minimal detectable changes index.

	Test μ	Retest μ			
	$\bar{x}$ (*S*D)	$\bar{x}$ (*S*D)	ICC	MDC95	MDC95_*es*_
Flex.MaxRange (°)	93.03 (15.59)	91.35 (15.98)	0.94	10.79	0.68
Flex.Max (°)	64.78 (10.32)	64.60 (10.60)	0.94	7.09	0.68
Flex.MaxMean (°)	63.06 (10.67)	62.74 (10.42)	0.95	6.77	0.64
Flex.MaxCV (%)	2.16 (1.36)	2.12 (1.26)	0.086	1.37	1.05
Flex.Min (°)	−28.48 (8.87)	−27.12 (9.16)	0.89	8.23	0.91
Flex.MinMean (°)	−25.37 (8.20)	−24.19 (8.87)	0.85	9.09	1.06
Flex.MinCV (%)	−9.44 (4.26)	−9.66 (5.42)	0.57	8.82	1.81
Flex.Speed.MaxMean (°/s) *	110.38 (17.32)	110.67 (22.59)	0.85	21.30	1.06
Flex.Speed.MinMean (°/s)	−89.47 (18.65)	−88.76 (17.60)	0.88	17.66	0.97
Flex.Speed.AreaMean (°/s)	216.42 (54.87)	217.98 (75.79)	0.87	66.49	1.00
Flex.Speed.AreaStd (°/s)	122.64 (82.01)	106.47 (46.29)	0.55	123.92	1.86
Flex.Speed.AreaCoef (%)	55.05 (30.81)	50.66 (22.88)	−0.11	79.41	2.93
Flex.SpeedUp.MaxMean (°/s)	482.02 (144.35)	506.80 (122.37)	0.91	109.47	0.82
Flex.SpeedUp.MinMean (°/s)	−494.68 (157.85)	−564.66 (163.66)	0.60	282.67	1.76
Flex.SpeedUp.Harmony (°/s)	−0.52 (0.12)	−0.51 (0.08)	0.59	0.18	1.79
Flex.SpeedUp.Offset (°/s)	115.74 (6.26)	115.23 (5.92)	0.75	8.48	1.39

Flex: flexion–extension movement; μ: mean; SD: standard deviation; ICC: intraclass correlation coefficient; MDC95_es: minimal detectable change in dimensionless value effect size at 95%; MDC95: minimal detectable change in absolute value at 95%. Parameters that showed significant differences between groups were marked with an asterisk (*).

**Table 4:** Test-retest results from rotation movement tests. Minimal detectable changes index.

	Test μ	Retest μ			
	$\bar{x}$ (*S*D)	$\bar{x}$ (*S*D)	ICC	MDC95	MDC95_*es*_
Rot.MaxRange (°)	82.03 (11.24)	81.35 (12.88)	0.93	8.65	0.72
Rot.Max (°)	41.03 (5.73)	40.05 (6.96)	0.87	6.26	0.98
Rot.MaxMean (°)	38.90 (5.65)	38.14 (6.69)	0.89	5.73	0.93
Rot.MaxCV (%)	4.12 (2.31)	3.80 (1.87)	0.42	4.44	2.11
Rot.Min (°)	−41.93 (6.93)	−41.60 (7.17)	0.88	6.69	0.95
Rot.MinMean (°)	−39.92 (6.64)	−39.10 (6.86)	0.94	4.46	0.66
Rot.MinCV (%)	−3.73 (2.40)	−4.50 (4.28)	0.58	6.20	1.79
Rot.Speed.MaxMean (°/s)	85.37 (20.82)	83.58 (21.51)	0.91	17.16	0.81
Rot.Speed.MinMean (°/s)	−82.53 (20.00)	−81.81 (21.24)	0.94	14.14	0.69
Rot.Speed.AreaMean (°/s)	166.40 (59.04)	161.89 (64.08)	0.94	40.76	0.66
Rot.Speed.AreaStd (°/s)	91.87 (42.67)	88.44 (41.59)	0.07	112.75	2.68
Rot.Speed.AreaCoef (%)	59.38 (30.81)	59.16 (28.92)	0.36	64.72	2.21
Rot.SpeedUp.MaxMean (°/s)	435.34 (105.80)	467.90 (107.38)	0.33	241.20	2.26
Rot.SpeedUp.MinMean (°/s)	−474.21 (142.97)	−447.03 (121.93)	0.19	330.98	2.49
Rot.SpeedUp.Harmony (°/s)	−0.52 (0.13)	−0.50 (0.13)	0.78	0.16	1.29
Rot.SpeedUp.Offset (°/s)	121.49 (9.03)	119.88 (8.47)	0.78	11.50	1.31

Rot: rotation movement; μ: mean; SD: standard deviation; ICC: intraclass correlation coefficient; MDC95_es: minimal detectable change in dimensionless value effect size at 95%; MDC95: minimal detectable change in absolute value at 95%.

**Table 5:** Test-retest results from lateralization movement tests. Minimal detectable changes index.

	Test μ	Retest μ			
	$\bar{x}$ (*S*D)	$\bar{x}$ (*S*D)	ICC	MDC95	MDC95_*es*_
Lat.MaxRange (°)	82.20 (9.97)	84.88 (9.29)	0.93	7.00	0.73
Lat.Max (°)	42.62 (6.06)	44.32 (5.30)	0.92	4.43	0.78
Lat.MaxMean (°)	41.16 (5.83)	42.16 (5.83)	0.94	3.70	0.67
Lat.MaxCV (%)	2.93 (1.62)	3.86 (2.82)	0.25	5.53	2.40
Lat.Min (°)	−39.58 (5.12)	−40.56 (5.50)	0.87	5.32	1.00
Lat.MinMean (°)	−37.90 (5.10)	−38.64 (5.09)	0.95	3.00	0.59
Lat.MinCV (%)	−3.48 (2.64)	−3.53 (3.10)	−0.03	8.09	0.59
Lat.Speed.MaxMean (°/s)	70.91 (17.15)	71.98 (16.46)	0.91	14.09	0.84
Lat.Speed.MinMean (°/s)	−73.19 (17.34)	−71.98 (16.46)	0.91	14.16	0.83
Lat.Speed.AreaMean (°/s)	150.35 (55.01)	156.26 (53.89)	0.95	32.90	0.60
Lat.Speed.AreaStd (°/s)	73.14 (31.86)	74.77 (35.00)	0.44	69.57	2.08
Lat.Speed.AreaCoef (%)	53.18 (27.17)	51.76 (24.60)	0.53	49.05	1.89
Lat.SpeedUp.MaxMean (°/s)	356.25 (91.40)	360.27 (100.21)	0.35	213.94	2.23
Lat.SpeedUp.MinMean (°/s)	−384.04 (106.50)	−355.99 (98.21)	0.38	223.91	2.19
Lat.SpeedUp.Harmony (°/s)	−0.55 (0.15)	−0.53 (0.15)	0.68	0.23	1.56
Lat.SpeedUp.Offset (°/s)	123.58 (10.18)	122.24 (10.44)	0.67	16.31	1.58

Lat: lateralization movement; μ: mean; SD: standard deviation; ICC: intraclass correlation coefficient; MDC95_es: minimal detectable change in dimensionless value effect size at 95%; MDC95: minimal detectable change in absolute value at 95%.

**Table 6:** Results of study for flexion–extension movement with deep oscillation treatment.

	Value Pre	Value Post	Mean dif	±MDC	N/U/P
Flex.DO.MaxRange (°)	64.26	80.32	16.06	2.79	0/0/100
Flex.DO.Max (°) *	51.46	61.72	10.25	1.83	0/0/100
Flex.DO.MaxMean (°) *	49.19	59.88	10.69	1.75	0/0/100
Flex.DO.MaxCV (%) *	3.51	2.36	−1.16	0.35	90/09/01
Flex.DO.Min (°) *	−12.79	−18.60	−5.81	2.12	95/05/0
Flex.DO.MinMean (°)	−10.08	−16.17	−6.08	2.35	97/03/0
Flex.DO.MinCV (%)	−22.16	−17.89	4.28	2.28	34/11/55
Flex.DO.Speed.MaxMean (°/s) *	77.41	103.41	26.01	5.50	0/0/100
Flex.DO.Speed.MinMean (°/s) *	−68.59	−89.53	−20.95	4.56	100/0/0
Flex.DO.Speed.AreaMean (°/s) *	116.34	195.13	78.79	17.17	0/0/100
Flex.DO.Speed.AreaStd (°/s)	53.62	69.27	15.65	32.00	0/100/0
Flex.DO.Speed.AreaCoef (%)	54.35	37.61	−16.75	20.50	31/69/0
Flex.DO.SpeedUp.MaxMean (°/s) *	360.73	437.13	76.40	28.27	0/06/94
Flex.DO.SpeedUp.MinMean (°/s)	−387.66	−472.13	−84.46	72.99	61/39/0
Flex.DO.SpeedUp.Harmony (°/s)	−0.57	−0.60	−0.03	0.05	37/60/03
Flex.DO.SpeedUp.Offset (°/s)	114.59	117.19	2.60	2.19	04/40/56

Flex: flexion–extension movement; DO: deep oscillation; MDC: minimal detectable change threshold; N: probability of negative changes; U: probability of unknown/trivial changes; P: probability of positive changes.. Parameters that showed significant differences between groups were marked with an asterisk (*).

**Table 7:** Results of study for rotation movement with deep oscillation treatment.

	Value Pre	Value Post	Mean dif	±MDC	N/U/P
Rot.DO.MaxRange (°) *	63.64	75.20	11.56	2.23	0/0/100
Rot.DO.Max (°) *	30.59	37.59	7.00	1.62	0/0/100
Rot.DO.MaxMean (°) *	28.58	35.94	7.37	1.48	0/0/100
Rot.DO.MaxCV (%)	6.25	3.98	−2.27	1.15	93/07/0
Rot.DO.Min (°) *	−33.06	−37.61	−4.55	1.73	98/02/0
Rot.DO.MinMean (°) *	−31.10	−35.71	−4.61	1.15	99/01/0
Rot.DO.MinCV (%)	−5.10	−4.37	0.72	1.60	0/84/16
Rot.DO.Speed.MaxMean (°/s) *	70.08	87.03	16.95	4.43	0/0/100
Rot.DO.Speed.MinMean (°/s) *	−69.55	−94.09	−24.55	3.65	100/0/0
Rot.DO.Speed.AreaMean (°/s) *	115.47	181.37	65.90	10.52	0/0/100
Rot.DO.Speed.AreaStd (°/s)	51.55	68.28	16.73	29.11	0/99/01
Rot.DO.Speed.AreaCoef (%)	48.39	41.32	−7.07	16.71	0/100/0
Rot.DO.SpeedUp.MaxMean (°/s) *	351.89	472.20	120.31	62.28	0/08/92
Rot.DO.SpeedUp.MinMean (°/s)	−371.03	−447.01	−75.98	85.46	41/59/0
Rot.DO.SpeedUp.Harmony (°/s)	−0.57	−0.64	−0.06	0.04	76/24/0
Rot.DO.SpeedUp.Offset (°/s)	124.96	129.07	4.11	2.97	0/30/70

Rot: rotation movement; DO: deep oscillation; MDC: minimal detectable change threshold; N: probability of negative changes; U: probability of unknown/trivial changes; P: probability of positive changes. Parameters that showed significant differences between groups were marked with an asterisk (*).

**Table 8:** Results of study for lateralization movement with deep oscillation treatment.

	Value Pre	Value Post	Mean dif	±MDC	N/U/P
Lat.DO.MaxRange (°) *	61.69	74.96	13.27	1.81	0/0/100
Lat.DO.Max (°) *	31.98	37.97	5.99	1.14	0/0/100
Lat.DO.MaxMean (°) *	30.70	36.80	6.10	0.96	0/0/100
Lat.DO.MaxCV (%)	3.75	2.79	−0.97	1.43	19/81/0
Lat.DO.Min (°) *	−29.71	−36.99	−7.28	1.37	100/0/0
Lat.DO.MinMean (°) *	−28.59	−36.00	−7.41	0.77	100/0/0
Lat.DO.MinCV (%)	−3.81	−2.34	1.46	2.09	0/70/30
Lat.DO.Speed.MaxMean (°/s) *	58.93	77.67	18.74	3.64	0/0/100
Lat.DO.Speed.MinMean (°/s) *	−61.35	−82.58	−21.23	3.66	100/0/0
Lat.DO.Speed.AreaMean (°/s) *	109.71	170.13	60.42	8.49	0/0/100
Lat.DO.Speed.AreaStd (°/s)	38.45	51.01	12.56	17.96	0/90/10
Lat.DO.Speed.AreaCoef (%)	43.31	35.54	−7.77	12.66	20/80/0
Lat.DO.SpeedUp.MaxMean (°/s) *	269.18	355.10	85.92	55.24	0/18/82
Lat.DO.SpeedUp.MinMean (°/s)	−278.67	−362.77	−84.10	57.81	82/18/0
Lat.DO.SpeedUp.Harmony (°/s)	−0.67	−0.71	−0.03	0.06	14/85/01
Lat.DO.SpeedUp.Offset (°/s)	132.43	135.13	2.70	4.21	01/76/23

Lat: lateralization movement; DO: deep oscillation; MDC: minimal detectable change threshold; N: probability of negative changes; U: probability of unknown/trivial changes; P: probability of positive changes. Parameters that showed significant differences between groups were marked with an asterisk (*).

**Table 9:** Results of study for flexion–extension movement with traditional treatment.

	Value Pre	Value Post	Mean dif	±MDC	N/U/P
Flex.TT.MaxRange (°) *	72.31	85.05	12.74	2.79	0/0/100
Flex.TT.Max (°) *	54.00	66.15	12.15	1.83	0/0/100
Flex.TT.MaxMean (°) *	51.09	64.20	13.11	1.75	0/0/100
Flex.TT.MaxCV (%) *	4.74	2.63	−2.11	0.35	100/0/0
Flex.TT.Min (°)	−18.30	−18.90	−0.60	2.12	23/67/10
Flex.TT.MinMean (°)	−16.11	−15.75	0.37	2.35	11/69/20
Flex.TT.MinCV (%) *	−10.61	−18.75	−8.13	2.28	62/8/30
Flex.TT.Speed.MaxMean (°/s) *	76.57	101.12	24.55	5.50	0/0/100
Flex.TT.Speed.MinMean (°/s) *	−71.44	−91.20	−19.76	4.56	100/0/0
Flex.TT.Speed.AreaMean (°/s) *	130.87	195.99	65.12	17.17	0/0/100
Flex.TT.Speed.AreaStd (°/s)	53.74	777.77	24.04	32.00	0/87/13
Flex.TT.Speed.AreaCoef (%)	59.27	52.44	−6.82	20.50	8/92/0
Flex.TT.SpeedUp.MaxMean (°/s) *	320.41	447.90	127.49	28.27	0/3/97
Flex.TT.SpeedUp.MinMean (°/s)	−324.74	−459.96	−135.23	72.99	92/8/0
Flex.TT.SpeedUp.Harmony (°/s)	−0.58	−0.62	−0.04	0.05	33/67/0
Flex.TT.SpeedUp.Offset (°/s)	117.97	118.46	0.48	2.19	0/90/10

Flex: flexion–extension movement; TT: traditional treatment; MDC: minimal detectable change threshold; N: probability of negative changes; U: probability of unknown/trivial changes; P: probability of positive changes. Parameters that showed significant differences between groups were marked with an asterisk (*).

**Table 10:** Results of study for rotation movement with traditional treatment.

	Value Pre	Value Post	Mean dif	±MDC	N/U/P
Rot.TT.MaxRange (°) *	69.42	77.84	8.42	2.23	0/0/100
Rot.TT.Max (°)	33.78	38.51	4.73	1.62	0/27/73
Rot.TT.MaxMean (°)	31.94	37.21	5.27	1.48	0/14/86
Rot.TT.MaxCV (%)	4.46	2.93	−1.52	1.15	75/25/0
Rot.TT.Min (°) *	−35.64	−39.33	−3.69	1.73	87/13/0
Rot.TT.MinMean (°) *	−33.65	−37.42	−3.77	1.15	92/8/0
Rot.TT.MinCV (%)	−4.95	−3.76	1.18	1.60	0/70/30
Rot.TT.Speed.MaxMean (°/s) *	73.33	90.45	17.12	4.43	0/0/100
Rot.TT.Speed.MinMean (°/s) *	−70.86	−90.90	−20.04	3.65	100/0/0
Rot.TT.Speed.AreaMean (°/s) *	126.99	185.66	58.67	10.52	0/0/100
Rot.TT.Speed.AreaStd (°/s)	52.05	68.55	16.50	29.11	0/100/0
Rot.TT.Speed.AreaCoef (%)	43.21	37.21	−6.00	16.71	0/100/0
Rot.TT.SpeedUp.MaxMean (°/s)	369.28	460.78	91.50	62.28	0/20/80
Rot.TT.SpeedUp.MinMean (°/s)	−349.55	−440.21	−90.66	85.46	60/40/0
Rot.TT.SpeedUp.Harmony (°/s)	−0.59	−0.67	−0.08	0.04	93/7/0
Rot.TT.SpeedUp.Offset (°/s)	126.58	132.75	6.17	2.97	0/5/95

Rot: rotation movement; TT: traditional treatment; MDC: minimal detectable change threshold; N: probability of negative changes; U: probability of unknown/trivial changes; P: probability of positive changes. Parameters that showed significant differences between groups were marked with an asterisk (*).

**Table 11:** Results of study for lateralization movement with traditional treatment.

	Value Pre	Value Post	Mean dif	±MDC	N/U/P
Lat.TT.MaxRange (°) *	60.25	68.09	7.84	1.81	0/5/95
Lat.TT.Max (°) *	30.74	35.48	4.74	1.14	0/3/97
Lat.TT.MaxMean (°) *	29.46	34.08	4.61	0.96	0/3/97
Lat.TT.MaxCV (%)	3.97	3.40	−0.57	1.43	4/96/0
Lat.TT.Min (°)	−29.51	−32.62	−3.11	1.37	84/16/0
Lat.TT.MinMean (°) *	−27.73	−31.38	−3.65	0.77	95/4/1
Lat.TT.MinCV (%)	−4.97	−3.30	1.68	2.09	0/74/26
Lat.TT.Speed.MaxMean (°/s) *	55.73	67.91	12.18	3.64	0/0/100
Lat.TT.Speed.MinMean (°/s) *	−54.56	−68.37	−13.81	3.66	100/0/0
Lat.TT.Speed.AreaMean (°/s) *	88.84	127.80	38.96	8.49	0/0/100
Lat.TT.Speed.AreaStd (°/s)	30.67	36.87	6.20	17.96	0/100/0
Lat.TT.Speed.AreaCoef (%)	35.39	29.76	−5.62	12.66	0/100/0
Lat.TT.SpeedUp.MaxMean (°/s)	244.55	291.50	46.95	55.24	0/68/32
Lat.TT.SpeedUp.MinMean (°/s)	−248.87	−282.96	−34.09	57.81	3/97/0
Lat.TT.SpeedUp.Harmony (°/s)	−0.67	−0.73	−0.06	0.06	54/46/0
Lat.TT.SpeedUp.Offset (°/s)	132.91	137.55	4.65	4.21	0/40/60

Lat: lateralization movement; TT: traditional treatment; MDC: minimal detectable change threshold; N: probability of negative changes; U: probability of unknown/trivial changes; P: probability of positive changes. Parameters that showed significant differences between groups were marked with an asterisk (*).

**Table 12:** Low-back-specific SF-36 Physical Functioning scale average scores and differences in both treatments.

	DOT Group	TT Group
Pre-Treatment	60.00	65.78
Post-treatment	74.67	75.67
Difference	14.67	9.89

## Data Availability

Data are contained within the article.

## Supplementary Materials

The following supporting information can be downloaded at: https://www.mdpi.com/article/10.3390/s24030913/s1, This article contains spreadsheet of the results of data collection (BMI, Ages, gender, pain intensity, and brief examination from both groups), Supplementary material S1; variables data collection and complete statistics tables from both groups, Supplementary material S2 and supplementary material S3; collection and complete statistics tables from the Healthy Group, supplementary material S4; and the results from the ow-back-specific Short Form (SF)-36 Physical Functioning scale from each patient, supplementary material S5.
